# Dual-Tree Complex Wavelet Transform and Image Block Residual-Based Multi-Focus Image Fusion in Visual Sensor Networks

**DOI:** 10.3390/s141222408

**Published:** 2014-11-26

**Authors:** Yong Yang, Song Tong, Shuying Huang, Pan Lin

**Affiliations:** 1 School of Information Technology, Jiangxi University of Finance and Economics, Nanchang 330032, China; E-Mail: tongsong91@126.com; 2 School of Software and Communication Engineering, Jiangxi University of Finance and Economics, Nanchang 330032, China; E-Mail: shuyinghuang2010@126.com; 3 Key Laboratory of Biomedical Information Engineering of Education Ministry, Institute of Biomedical Engineering, Xi'an Jiaotong University, Xi'an 710049, China; E-Mail: linpan@mail.xjtu.edu.cn

**Keywords:** multi-focus image fusion, dual-tree complex wavelet transform, image block residual, visual sensor networks

## Abstract

This paper presents a novel framework for the fusion of multi-focus images explicitly designed for visual sensor network (VSN) environments. Multi-scale based fusion methods can often obtain fused images with good visual effect. However, because of the defects of the fusion rules, it is almost impossible to completely avoid the loss of useful information in the thus obtained fused images. The proposed fusion scheme can be divided into two processes: initial fusion and final fusion. The initial fusion is based on a dual-tree complex wavelet transform (DTCWT). The Sum-Modified-Laplacian (SML)-based visual contrast and SML are employed to fuse the low- and high-frequency coefficients, respectively, and an initial composited image is obtained. In the final fusion process, the image block residuals technique and consistency verification are used to detect the focusing areas and then a decision map is obtained. The map is used to guide how to achieve the final fused image. The performance of the proposed method was extensively tested on a number of multi-focus images, including no-referenced images, referenced images, and images with different noise levels. The experimental results clearly indicate that the proposed method outperformed various state-of-the-art fusion methods, in terms of both subjective and objective evaluations, and is more suitable for VSNs.

## 1. Introduction

Visual sensor networks (VSNs), wherein camera-equipped sensor nodes can capture, process, and transmit visual information, are an important research area which has attracted considerable attention in recent years [1,2]. Camera sensors collect a huge amount of visual data, which are rich in information and offer tremendous potential when used in VSNs [3,4]. Due to the resource and bandwidth requirements in VSNs, how to process such data using generally low-power sensor nodes is becoming a problem [5,6]. Image fusion can fuse these pictures into a single image. The fused image has a complete description of the scene, and is more accurate and suitable for both visual perception and further processing. This processing can reduce the randomness and redundancy and save storage space and bandwidth on the network, and hence improve the transmission efficiency in VSNs [7].

Because of the limited depth of focus in optical lenses [8], it is difficult to acquire an image that contains all relevant focused objects and hence some objects are in focus, but others will be out of focus and, thus, blurred. In VSNs, we have the opportunity to extend the depth of focus by using image fusion techniques.

Recently, image fusion techniques have been performed either in the spatial domain or frequency domain [9]. Spatial domain techniques are carried out directly on the source images. The main spatial domain techniques are weighted average, principal component analysis (PCA), intensity-hue-saturation (IHS), and Brovey transform [10]. The fused images obtained by these methods have high spatial quality, but they usually overlook the high quality of spectral information and hence suffer from spectral degradation [11]. In 2002, Li *et al.* [12] introduced the artificial neural network (ANN) for use in multi-focus image fusion. However, the performance of the ANN depends on the sample images and this is not an appealing characteristic. In 2011, Tian *et al.* [13] proposed a bilateral gradient to perform image fusion. However, there were some erroneous selections of some blocks in the focus region due to noise or undesired effects. Since the actual objects usually contain structures at many different scales or resolutions and multi-scale techniques are similar to the human visual system (HVS), the multi-scale techniques have attracted increasing interest in image fusion [14].

So far, frequency domain methods have been explored by using multi-scale transforms, including pyramid transforms [15,16], wavelet transforms [17,18], fast discrete curvelet transform (FDCT) [19], complex wavelet transform (CWT) [20] and non-subsampled contourlet transform (NSCT) [21]. Due to the outstanding localization capability in both the time and frequency domains, wavelet analysis has become one of the most commonly used methods in frequency domain fusion [18]. However, wavelet analysis cannot represent the directions of the image edges accurately, which results in a lack of shift-invariance and the appearance of pseudo-Gibbs phenomena in the fused image [22]. To overcome these shortcomings of the wavelet transform, Da Cunha *et al.* [23] proposed the NSCT. This method can not only give the asymptotic optimal representation of contours, but also possesses shift-invariance, and effectively suppresses pseudo-Gibbs phenomena [24]. In 2008, Qu *et al.* [25] proposed an image fusion method based on spatial frequency-motivated pulse coupled neural networks (SF_PCNN) in the NSCT domain, and proved that this method could extract more useful information and provide much better performance than typical fusion methods. However, the NSCT-based algorithm is time-consuming and of high complexity. The dual-tree complex wavelet transform (DTCWT) is also proposed for image fusion. This method deals with the shift variance phenomenon using parallel wavelet filtering with directionality support (six planes) [26]. Furthermore, this filtering provides a 1/2 sample delay in the wavelet branches of the dual trees allowing near-shift invariance and perfect reconstruction [27].

Multi-scale techniques can significantly enhance the visual effect, but in the focus area of the source image, the fused image clarity will have different degrees of information loss. This occurs because in the process of multi-scale decomposition and reconstruction, improper selection of fusion rules often causes the loss of useful information in the source image; this defect is almost impossible to completely avoid in the multi-scale based image fusion method [9].

In addition, it is noteworthy that there is a hypothesis whereby the source images are noise-free in most of the image fusion methods. These methods can always produce high-quality fused images with this assumption [28]. However, the images are often corrupted by noise that cannot be completely avoided during the image acquisition from VSNs [28].

Based on the above analysis, an image fusion method based on DTCWT with image block residual is proposed in this paper. The main differences and advantages of the proposed framework compared to other methods can be summarized as follows:

(1) For VSNs, a novel multi-focus image fusion framework which combines the DTCWT and image block residual techniques is proposed. The framework is divided into visual contrast based DTCWT based initial fusion and block residual based final fusion processes.
(2) In the visual contrast based DTCWT-based initial fusion process, the Sum-Modified-Laplacian (SML)-based visual contrast [29] and SML [30] are employed as the rules for low- and high-frequency coefficients in DTCWT domain, respectively. Using this model, the most important feature information is selected in the fused coefficients.
(3) In the block residual-based final fusion process, the image block residuals technique and consistency verification are proposed to detect the focus area and then a decision map is obtained. The decision map is used to guide which block should be selected from the source images or the initial fused image. Using this model, we can select pixels from the focus areas of source images to avoid loss of useful information to the greatest extent.
(4) Further, the proposed framework can provide a better performance than the various state-of-the-art fusion methods and is robust to noise. In addition, the proposed framework is efficient and more suitable for VSNs.

The rest of the paper is organized as follows: the related theories of the proposed framework are introduced in Section 2. The proposed fusion framework is described in Section 3. Experimental results and analysis are given in Section 4, and the concluding remarks are described in Section 5.

## 2. Preliminaries

This section covers two related concepts, including DTCWT and SML-based visual contrast, as described below.

### 2.1. Dual-Tree Complex Wavelet Transform (DTCWT)

**Figure 1.**
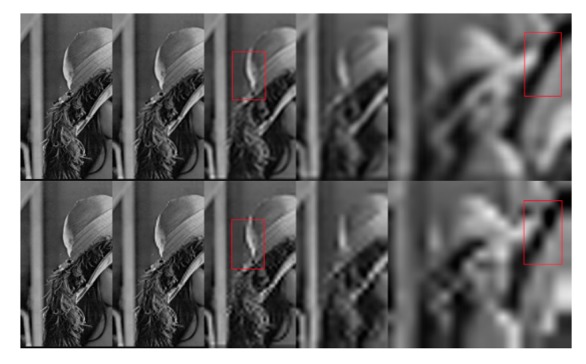
Accumulated reconstructions from each level of DTCWT (**top**) and DWT (**bottom**).

Wavelet transforms provide a framework in which an image is decomposed, with each level corresponding to a coarser resolution band [17]. Once the wavelet transform is implemented, every second wavelet coefficient at each decomposition level is discarded, resulting in components that are highly dependent on their location in the subsampling vector and with great uncertainty as to when they occurred in time [27]. This unfavorable property is referred to as shift variance (throwing away 1 of every 2 samples). Coupled with the limitations in the direction, wavelet analysis thus cannot accurately represent the directions of the edges of images [22]. To overcome these shortcomings of DWT, in 1998 Kingsbury [26] proposed the dual-tree complex wavelet transform (DTCWT), an over-complete wavelet transform which provides both good shift invariance and directional selectivity. The DTCWT idea is based on the use of two parallel trees, one for the odd samples and the other one for the even samples generated at the first level. These trees provide the signal delays necessary for every level and hence eliminate aliasing effects and achieve shift invariance [31]. The horizontal and vertical sub-bands are divided into six distinct sub-bands which are ±15, ±45, ±75. The advantages of DTCWT, *i.e.*, shift invariance and directional sensitivity, give improved fusion results that outperform the discrete wavelet transform (DWT) [32]. Figure 1 shows the accumulated reconstructions from each level of DTCWT and DWT after four levels of decomposition. As shown the edge of the decomposed image (highlighted in the red rectangle), pseudo-Gibbs phenomena appear in DWT, while DTCWT shows smooth edges. That is because DTCWT can represent the line singularities and plane singularities more effectively than DWT. Detailed information about the design of DTCWT may be found in [31].

### 2.2. Sum-Modified-Laplacian Based Visual Contrast

For spatial domain based multi-focus image fusion, there are many typical focus measurements such as energy of image gradient (EOG), spatial frequency (SF), tenengrad, Laplacian energy and SML. In [33], the authors compared these measurements through extensive experiments, and the results proved that SML is the best measurement. In the transform domain, SML is also very efficient and can produce the best fused results [34]. The definition of SML is as follows:

(1) $$\text{SML}(i,j)=\overset{M_{1}}{\underset{m=-M_{1}}{\text{∑}}}\overset{N_{1}}{\underset{n=-N_{1}}{\text{∑}}}\left[ML(i+m,j+n)\right],$$

where:

(2) $$\begin{array}{l} \begin{array}{ll} ML(i,j) & =\left|2L(i,j)-L(i-\text{step},j)-L(i+\text{step},j)\right|\\ & +\left|2L(i,j)-L(i,j-\text{step})-L(i,j+\text{step})\right|, \end{array} \end{array}$$

where (2*M*_1_ + 1)(2*N*_1_ + 1) is the size of the window, *step* is a variable spacing between coefficients and always equal to 1 [33]. *L*(*i*, *j*) denotes the pixel value of one coefficient located at (*i*, *j*). According to physiological and psychological research, the human visual system is highly sensitive to the local contrast of the image rather than the value of the pixel [35]. To meet this requirement, local visual contrast is proposed [36]. Considering the excellent clear measurement of SML, the visual contrast based on SML [30] is introduced in this article. This scheme is defined as:

(3) $$VC_{\text{SML}}(i,j)=\{\begin{array}{ll} \frac{\text{SML}(i,j)}{\hat{L}{\left(i,j\right)}^{1+\alpha }},if & \hat{L}(i,j)\neq 0\\ \text{SML}(i,j), & \text{otherwise} \end{array},$$

where α is a visual constant representing the slope of the best-fitted lines through high-contrast data, which is determined by physiological vision experiments, and it ranges from 0.6 to 0.7 [34]. *L̂*(*I*, *j*) is the mean intensity value of the pixel (*i*, *j*) centered of the neighborhood window.

## 3. The Proposed Image Fusion Method

**Figure 2.**
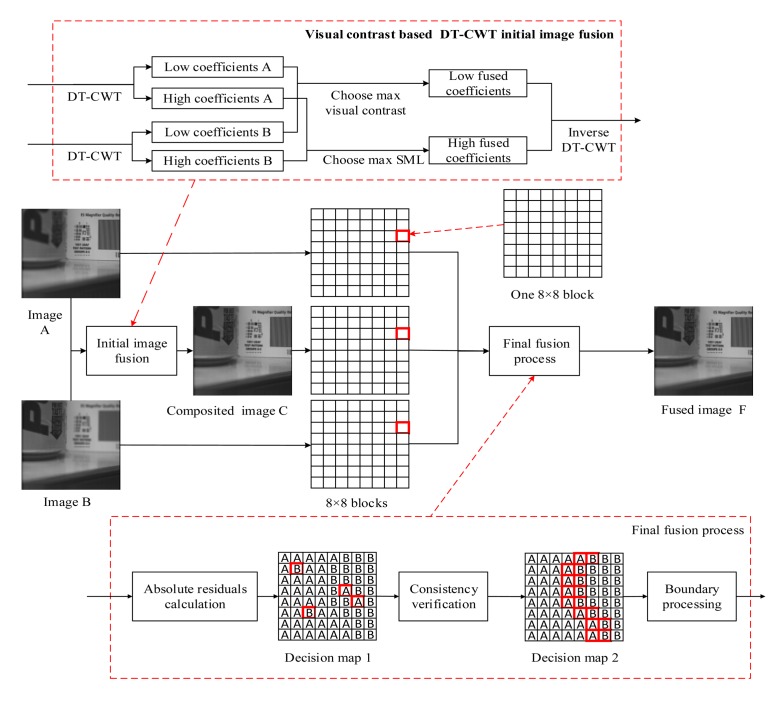
The framework of the proposed fusion scheme.

In this section, the proposed image fusion framework is depicted in Figure 2.

The framework includes two fusion procedures: the DTCWT based initial image fusion process and a hybrid final fusion process. The two processes are described as follows.

### 3.1. DTCWT Based Initial Image Fusion

In this subsection, the DTCWT based initial fusion, which applies new fusion rules for low- and high-frequency coefficients, respectively, is described in Algorithm 1.



**Algorithm 1.** DTCWT based initial image fusion
**Input:** Source images.**Step 1:** Load the source multi-focus images.**Step 2:** Perform three levels (how to set the level can be seen in literature [32]) DTCWT on source images to obtain two low frequency sub-bands and six high frequency sub-bands at each level. These sub-bands are denoted as:
(4)$$\left\{\left(L_{t}^{X},H_{l,d}^{X}\right)|X=A,B;l=1,2,3;t=1,2;d=1,\ldots ,6\right\},$$where 
$L_{t}^{X}$ is the low frequency sub-bands in the *t* orientation, 
$H_{l,d}^{X}$ represent the high frequency sub-bands *l* level in the *d* orientation.**Step 3:** Fuse low- and high-frequency sub-bands via the following different rules to obtain composite low- and high-frequency sub-bands. The detail of low coefficients and high coefficients fusion are discussed as follows.**Step 4:** Perform three levels inverse DTCWT on the composited low- and high-frequency sub-bands to obtain the composited image.**Output:** The initial composited image.


The low frequency coefficients represent the approximate information and contain the most energy of the source images. The widely used rule is to apply averaging methods to produce the fused coefficients. However, this rule will reduce the contrast in the fused images. According to [30], the SML-based visual contrast is a criterion which considers the nonlinear relationship between the contrast sensitivity threshold of HVS and the background luminance. Therefore, we introduce SML-based visual contrast to fuse the low frequency sub-bands and the fusion process is given as follows:

(5) $$L_{t}^{C}(i,j)=\{\begin{array}{ll} L_{t}^{A}(i,j), & if\;VC_{{\text{SML}}_{t}}^{A}(i,j)\geq VC_{{\text{SML}}_{t}}^{B}(i,j)\\ L_{t}^{B}(i,j), & \text{otherwise} \end{array},$$

where $VC_{{\text{SML}}_{t}}^{A}(i,j)$ and $VC_{{\text{SML}}_{t}}^{B}(i,j)$ are the visual contrast extracted from low frequency sub-bands at the *t*-th direction of the corresponding source image.

For the high frequency coefficients, the most popular fusion rule is to select the coefficients with larger absolute values, but this rule does not take any consideration of the surrounding pixels. The SML operator is developed to provide local measures of the quality of image focus [29]. In [33], it is proved that the SML is very efficient in the transform domain. Therefore, the composite rule for the high-frequency sub-bands is defined as:

(6) $$H_{l,d}^{C}(i,j)=\{\begin{array}{ll} H_{l,d}^{A}(i,j), & if\;{\text{SML}}_{l,d}^{A}(i,j)\geq {\text{SML}}_{l,d}^{B}(i,j)\\ H_{l,d}^{B}(i,j), & \text{otherwise} \end{array},$$

where ${\text{SML}}_{l,d}^{A}\left(i,j\right)$ and ${\text{SML}}_{l,d}^{B}\left(i,j\right)$ represent the SML extracted from high frequency sub-bands at the *l*-th scale and *d*-th direction of the corresponding source image.

### 3.2. The Final Fusion Process

After DTCWT-based initial image fusion, a novel hybrid method for obtaining the final fused image is proposed and the final fusion process is described in Algorithm 2. The detailed description of some key techniques and algorithms is as follows.



**Algorithm 2.** The final fusion process
**Input:** The source images and the initial composited image.**Step 1:** Load the source images and the initial composited image.**Step 2:** The source images and the initial fused image are partitioned into non-overlapping blocks.**Step 3:** Calculate the values of image block residual between the source images and initial composited image.**Step 4:** Compare the values of *RV^A^*(*i*_0_, *j*_0_) and *RV^B^*(*i*_0_, *j*_0_) (block residuals of A and B), and then obtain an initial decision map *M*_1_(*i*_0_, *j*_0_).**Step 5:** Consistency verification is employed to eliminate the defects of *M*_1_(*i*_0_, *j*_0_) that caused by noise or undesired effects, and then get a modified decision map *CV*(*i*_0_, *j*_0_) for next step.**Step 6:** Get a final decision map *Z*(*i*_0_, *j*_0_) by boundary processing. The final process can thus be illustrated as follows:
(7)$$F(i_{0},j_{0})=\left\{ \begin{array}{l} A(i_{0},j_{0}),if\;Z(i_{0},j_{0})>0\\ B(i_{0},j_{0}),if\;Z(i_{0},j_{0})<0\\ C(i_{0},j_{0}),if\;Z(i_{0},j_{0})=0 \end{array}\right.,$$where *A*(*i*_0_, *j*_0_), *B*(*i*_0_, *j*_0_), *C*(*i*_0_, *j*_0_), *F*(*i*_0_, *j*_0_) denote the pixels of the block located at (*i*_0_, *j*_0_) of the source images, the composited image, and the final fused image, respectively.**Output:** The final fused image.


#### 3.2.1. Image Block Residual

In multi-focus images, the focused region is more informative [28]. It is easy to find that blocks in the focus area have greater similarity to the blocks of the initial fused image. Therefore, the image block residual is proposed to test the similarity, which is defined as:

(8) $$RV^{A}\left(i_{0},j_{0}\right)=\left|\overset{N_{0}i_{0}}{\underset{i=N_{0}i_{0}-N_{0}+1}{\text{∑}}}\overset{N_{0}j_{0}}{\underset{j=N_{0}j_{0}-N_{0}+1}{\text{∑}}}C{\left(i,j\right)}^{2}-\overset{N_{0}i_{0}}{\underset{i=N_{0}i_{0}-N_{0}+1}{\text{∑}}}\overset{N_{0}j_{0}}{\underset{j=N_{0}j_{0}-N_{0}+1}{\text{∑}}}A{\left(i,j\right)}^{2}\right|$$

(9) $$RV^{B}\left(i_{0},j_{0}\right)=\left|\overset{N_{0}i_{0}}{\underset{i=N_{0}i_{0}-N_{0}+1}{\text{∑}}}\overset{N_{0}j_{0}}{\underset{j=N_{0}j_{0}-N_{0}+1}{\text{∑}}}C{\left(i,j\right)}^{2}-\overset{N_{0}i_{0}}{\underset{i=N_{0}i_{0}-N_{0}+1}{\text{∑}}}\overset{N_{0}j_{0}}{\underset{j=N_{0}j_{0}-N_{0}+1}{\text{∑}}}B{\left(i,j\right)}^{2}\right|$$

where *N*_0_ × *N*_0_ is the size of the blocks (how to set the size of block can be seen in literature [5,7]). *A*(*i*, *j*), *B*(*i*, *j*), and *C*(*i*, *j*) are the pixel values of the source images and the composited image, respectively.

The block residual can be assumed as a similarity measure in image processing applications. Constructing an initial decision map by:

(10) $$M_{1}(i_{0},j_{0})=\{\begin{array}{ll} -1, & if\;RV^{A}(i_{0},j_{0})<RV^{B}(i_{0},j_{0})\\ 1, & \text{otherwise} \end{array}$$

#### 3.2.2. Consistency Verification

In a scene, we assume most blocks of a region are in the depth of focus of one source image. According to the theory of imaging, all the blocks in such region must be chosen from the image [5]. However, there can be some erroneous selection of some blocks due to noise or undesired effects. To remove these defects, consistency verification [37] is employed. If the center block comes from one image while the majority of the surrounding blocks come from the other one, the center sample is simply switched to the corresponding block in the other image. Here, consistency verification is applied in a 3 × 3 neighborhood window as in [7]. After consistency verification to *M*_1_(*i*_0_, *j*_0_), we get the modified decision map *CV*(*i*_0_, *j*_0_) for boundary processing.

#### 3.2.3. Boundary Processing

Assume one block contains both clear areas and blurred areas, so it is meaningless to judge the block is in the focus area or not. According to the theory of imaging, these blocks are always on the boundary of focused region. Therefore, we could choose these blocks by the equations below:

(11) $$\begin{array}{ll} Z(i_{0},j_{0}) & =\max\left(\left\{M_{2}(i_{0},j_{0}),M_{2}(i_{0}+1,j_{0}),M_{2}(i_{0},j_{0}+1),M_{2}(i_{0}+1,j_{0}+1)\right\}\right)\\ & +\min\left(\left\{M_{2}(i_{0},j_{0}),M_{2}(i_{0}+1,j_{0}),M_{2}(i_{0},j_{0}+1),M_{2}(i_{0}+1,j_{0}+1)\right\}\right), \end{array}$$

in which:

(12) $$M_{2}(i_{0},j_{0})=\{\begin{array}{ll} 1, & if\;CV(i_{0},j_{0})>0\\ -1, & \text{otherwise} \end{array}$$

If *M*_2_(*i*_0_, *j*_0_) = 1, the block at (*i*_0_, *j*_0_) from image A is from the focus area, and if *M*_2_(*i*_0_, *j*_0_) = −1, the block at (*i*_0_, *j*_0_) from image B is from the focus area. Equation (11) means if one of *M*_2_(*i*_0_, *j*_0_), *M*_2_(*i*_0_ + 1, *j*_0_), *M*_2_(*i*_0_, *j*_0_ + 1) and *M*_2_(*i*_0_ + 1, *j*_0_ + 1) equals to 1 and another one equals to −1, the block at (*i*_0_, *j*_0_) is on the boundary of focused region.

As we know, the initial fused image has extracted the most characteristics of the source images to some extent. On the boundary of the focused region, the blocks may contain both clear pixels and blurred pixels. To reduce the loss of useful information, we should choose the blocks of the initial fused image on the boundary of the focused region. Then, we can use the Equation (7) to get the fusion result.

## 4. The Experimental Results and Analysis

In this section, the evaluation index system and the experimental results and analysis are described.

### 4.1. Evaluation Index System

Generally speaking, the measure of image fusion quality can be divided into two stages: subjective metrics and objective metrics [38]. Subjective evaluations always depend on the human visual characteristics and the professional knowledge of observers, thus the processes are time-consuming and have poor repeatability [39]. Objective evaluations can be easily performed by computers, completely automatically, and generally evaluate the similarity between the fused and the referenced image or source images [40]. Therefore, besides visual observation, six objective performance metrics are used to demonstrate the superior performance of the proposed algorithm. The quantitative evaluations can be divided into two classes: objective evaluation of results of referenced images and objective evaluation of results of non-referenced images.

#### 4.1.1. Objective Evaluation of Results of Referenced Images

(1) *Root Mean Square Error (RMSE)*: The RMSE is the cumulative squared error between the fused image and the referenced image and it is defined by:
  (13) $$\text{RMSE}=\sqrt{\frac{\overset{M}{\underset{m=-M}{\text{∑}}}\overset{N}{\underset{n=-N}{\text{∑}}}{\left[F\left(i+m,j+n\right)-R\left(i+m,j+n\right)\right]}^{2}}{MN}}$$
  where *M* × *N* is the size of fused image, *F*(*i*, *j*) and *R*(*i*, *j*) are the pixel value of the fused image and referenced image at the position (*i*, *j*), respectively. The lower the value of RMSE, the better the fusion effect.
(2) *Peak Signal to Noise Ratio (PSNR)*: The PSNR is a ratio between the maximum possible power of a signal and the power of noise that affects the fidelity [41]. The PSNR is formulated as:
  (14) $$\text{PSNR}=10\mathrm{lg}\left|\frac{L^{2}}{{\text{RMSE}}^{2}}\right|$$
  where *L* is the dynamic range of the pixel values. Here, *L* = 255 is adopted. The greater the value of PSNR, the better the fusion effect.
(3) *Structural Similarity-based Metric (SSIM)*: The SSIM is designed by modeling any image distortion as the combination of the loss of correlation and radiometric and contrast distortion [42]. SSIM between the fused image and the referenced image is defined as follows:
  (15) $$\text{SSIM}\left(R,F\right)=\frac{\left[2u_{R}u_{F}-{\left(K_{1}L\right)}^{2}\right]\left[2\sigma_{RF}+{\left(K_{2}L\right)}^{2}\right]}{\left[{u_{R}}^{2}+{u_{F}}^{2}+{\left(K_{1}L\right)}^{2}\right]\left[{\sigma_{R}}^{2}+{\sigma_{F}}^{2}+{\left(K_{2}L\right)}^{2}\right]}$$
  in which *u_R_*, *u_F_* are mean intensity of the standard image and the fused image, respectively. σ*_R_*, σ*_F_*, σ*_RF_* are the variances and covariance, respectively [43]. *K*_1_ and *K*_2_ are small constants, and *L* is the dynamic range of the pixel values. Here, the default parameters: *K*_1_ = 0.01, *K*_2_ = 0.03, *L* = 255 is adopted [44]. The greater the value of SSIM, the better the fusion effect.

#### 4.1.2. Objective Evaluation of Results of Non-Referenced Images

(1) *Standard deviation:* The standard deviation can be used to estimate how widely spread the gray values in an image and defined as [45]:
  (16) $$\text{Std}=\sqrt{\left(\frac{1}{M\times N}\overset{M}{\underset{i=1}{\text{∑}}}\overset{N}{\underset{j=1}{\text{∑}}}{\left(F(i,j)-\hat{\mu }\right)}^{2}\right)},$$
  where μ̂ is the mean value of the fused image. The larger the standard deviation, the better the result.
(2) *Mutual Information (MI)*: The MI can indicate how much information the fused image conveys about the source images [33]. MI between fusion image and the source images is defined as follows [46]:
  (17) $$\text{MI}={\text{MI}}^{AF}+{\text{MI}}^{BF},$$
  in which:
  (18) $${\text{MI}}^{AF}=\overset{L}{\underset{f=0}{\text{∑}}}\overset{L}{\underset{a=0}{\text{∑}}}p^{AF}(a,f)\log_{2}\left(\frac{p^{AF}(a,f)}{p^{A}(a)p^{F}(f)}\right),$$
  (19) $${\text{MI}}^{BF}=\overset{L}{\underset{f=0}{\text{∑}}}\overset{L}{\underset{b=0}{\text{∑}}}p^{BF}(b,f)\log_{2}\left(\frac{p^{BF}(b,f)}{p^{B}(b)p^{F}(f)}\right),$$
  where MI*^AF^* and MI*^BF^* denote the normalized MI between the fused image and the source image A and B; *a*, *b* and *f* ε [0, L]. *P^A^*(*a*), *p^B^*(*B*) and *p^F^*(*f*) are the normalized gray level histograms of source images and fused image. *p^AF^*(*a*, *f*) and *p^BF^*(*b*, *f*) are the joint gray level histograms between the fused image and the source image A and B. The greater the value of MI, the better the fusion effect.
(3) *Edge Based Similarity Measure (Q^AB^*^/^*^F^)*: Q^AB/F^ which proposed by Xydeas and Petrovic [47] gives the similarity between the edges transferred from the source images to the fused image. The definition is given as:
  (20) $${\text{Q}}^{AB/F}=\frac{{\text{∑}}_{i=1}^{M}{\text{∑}}_{j=1}^{N}\left[{\text{Q}}^{AF}(i,j)w^{A}(i,j)+{\text{Q}}^{BF}(i,j)w^{B}(i,j)\right]}{{\text{∑}}_{i=1}^{M}{\text{∑}}_{j=1}^{N}\left[w^{A}(i,j)+w^{B}(i,j)\right]},$$
  in which:
  (21) $${\text{Q}}^{AF}(i,j)={\text{Q}}_{a}^{AF}(i,j)Q_{g}^{AF}(i,j),$$
  (22) $${\text{Q}}^{BF}(i,j)={\text{Q}}_{a}^{BF}(i,j){\text{Q}}_{g}^{BF}(i,j),$$
  where *w^A^*(*i*, *j*) and *w^B^*(*i*, *j*) are the corresponding gradient strengths for images A and B, respectively. $Q_{a}^{xF}(i,j)$ and $Q_{g}^{xF}(i,j)$ are the Sobel edge strength and orientation preservation values at location (*i*, *j*) for each source image [48]. The larger the value, the better the fusion effect result.

### 4.2. Experiments on Multi-Focus Image Fusion

The proposed fusion framework is tested on three sets of multi-focus image databases: non-referenced images, referenced images, and images with different noise. For all these image databases, the results of the proposed fusion scheme are compared with those of the state-of-the-art wavelet transform-, FDCT-, DTCWT-, and NSCT-based methods. The low- and high-frequency coefficients of the wavelet transform-, FDCT-, and DTCWT (DTCWT-1)-based methods are merged by the widely used fusion rule of averaging and selecting larger absolute values of the coefficient (average-maximum rule), respectively. The DTCWT-2 method is the DTCWT-based initial image fusion technique proposed in this paper. In addition, we introduce two NSCT-based methods (NSCT-1 and NSCT-2) for comparison. NSCT-1 involves merging the coefficients by an average-maximum rule; NSCT-2 merges the coefficients by SF_PCNN [25], which was proposed by Qu *et al.* in [25] and can make full use of the characteristics of PCNN and has been proven to outperform PCNN in the NSCT domain in fusing multi-focus images. In order to perform a fair comparison, the source images are all decomposed into three levels for those methods, except for the FDCT-based method (five is proven in [9] to be the best level in the FDCT-based image fusion method). For the wavelet-based method, the images are decomposed using the DBSS (2, 2) wavelet. For implementing NSCT, “9-7” filters and “pkva” filters (how to set the filters can be found in [49]) are used as the pyramidal and directional filters, respectively. It should be noted that since image registration is out of scope of this paper, like all the literatures, in all cases we assume the source images have been in perfect registration. A thorough survey of image registration techniques can be found in [50]. All the experiments are implemented in MATLAB R2012a on an Intel (R) Core (TM) i3-2330M CPU @2.2 GHz computer (ASUS, Taipei, Taiwan) with 4 GB RAM.

#### 4.2.1. Fusion of Referenced Multi-Focus Images

**Figure 3.**
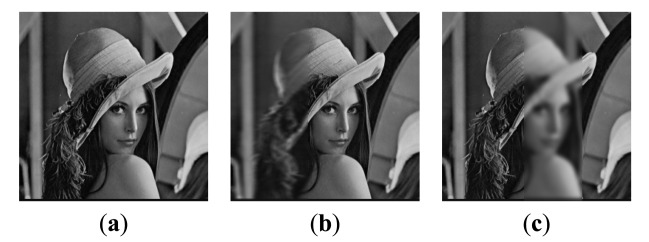
Referenced and blurred Lena images. (**a**) Referenced image; (**b**) Blurred on left; (**c**) Blurred in the middle.

**Figure 4.**
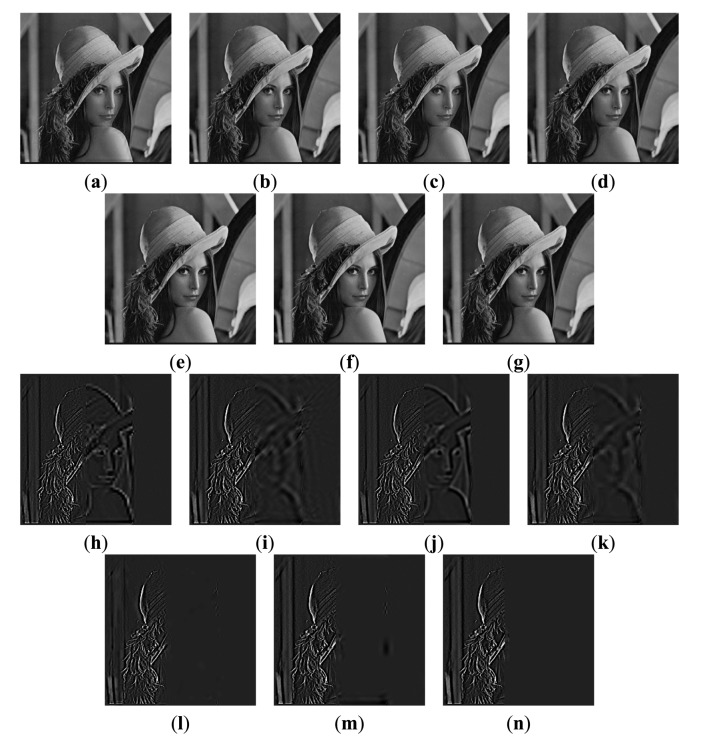
Fusion results of the Lena image. Fused images of: (**a**) Wavelet transform-based method; (**b**) FDCT-based method; **(c)** NSCT-1-based method; (**d**) DTCWT-1-based method; (**e**) NSCT-2-based method; (**f**) DTCWT-2-based method; (**g**) our proposed method; (**h**–**n**) the sharpened residual images between the seven results and Figure 3b.

The first experiment is conducted using an extensive set of artificially generated images. Figure 3 shows the referenced Lena image, and two blurred artificial images that are generated by convolving the referenced image with an averaging filter centered on the left part and the middle part, respectively [51]. The fusion results of different images are shown in Figure 4a–g. For a clearer comparison, this paper has given the residual images [28] between the results and Figure 3b, and performed the following transform on each pixel of each residual image:

(23) $$D^{*}(i,j)=3\times D(i,j)+50$$

where the *D*(*i*, *j*) is the pixel of residual image located at (*i*, *j*). Figure 4h–n shows the sharpened residual images.

For the focused areas, the residual values between the fused images and the source image should be zero. According to the literature [9], in residual images, the lower residue the features, the more detail information in the focus region has been transferred to fused images and the better the corresponding method. From Figure 4, as mentioned above, the residual image between the result of the proposed method and Figure 3b is almost zero in the clear region. That is to say, the proposed method has extracted the most information from the clear region of Figure 3b.

**Figure 5.**
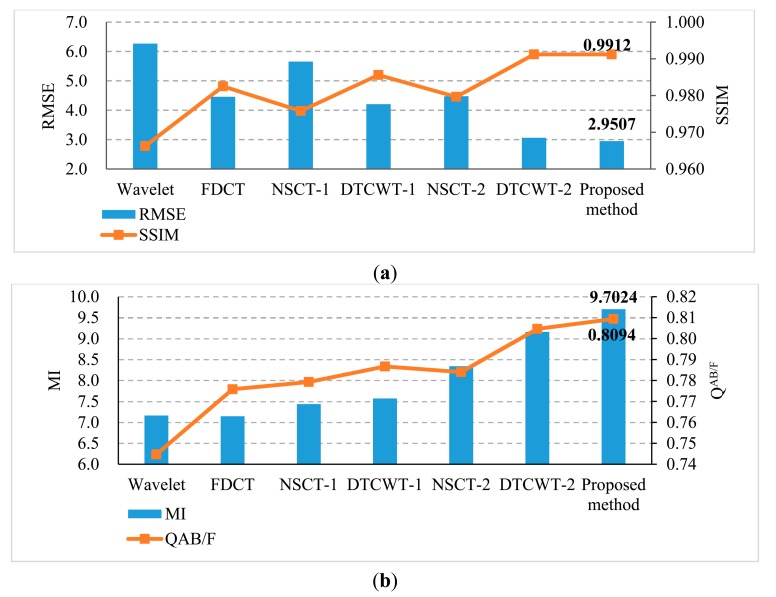
Comparison on (**a**) RMSE, SSIM and (**b**) MI, Q^AB/F^ of different methods for Lena image.

RMSE and SSIM are the metrics that measure the error or similarity between a referenced image and a fused image. Using these metrics the effect of the methods in the fusion of referenced multi-focus images can be measured well. In addition, MI and Q^AB/F^ are the state-of-the-art fusion performance metrics [25,40], which can be used to measure the fusion of no-reference multi-focus images. Figure 5 depicts the comparison on quantitative metrics of different methods; it is easy to find that the SSIM value of the proposed method is the largest (0.9912) and RMSE value is the smallest (2.9507). Both of them are the optimal values. That is to say, compared with other methods, the fused image of the proposed method is the closest to the referenced image. In addition, the MI and Q^AB/F^ values of the proposed method results also outperform the other methods.

**Figure 6.**
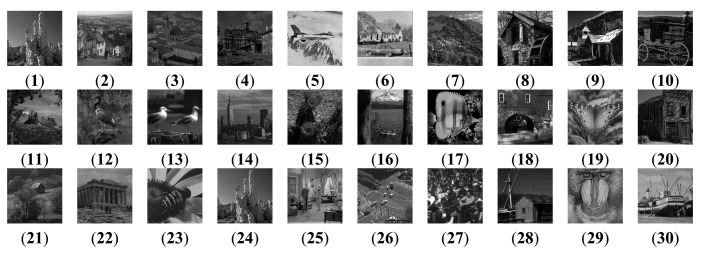
Standard gray test image database.

**Figure 7.**
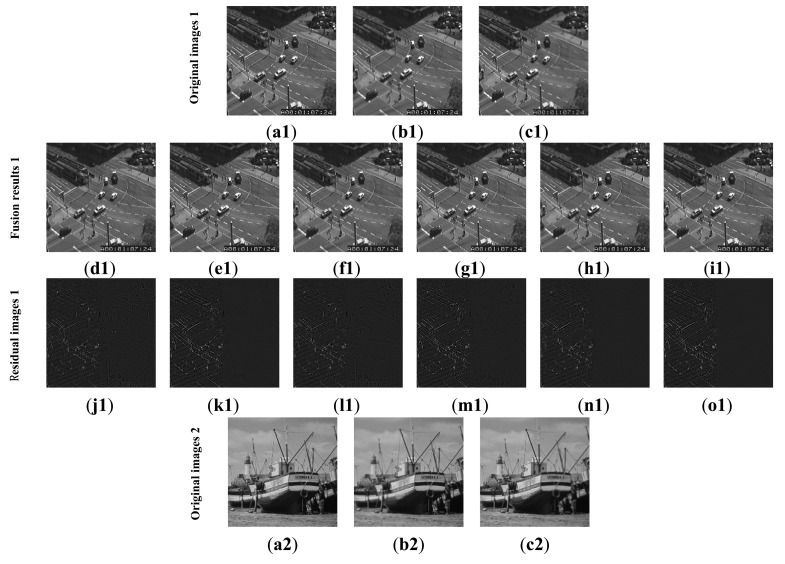

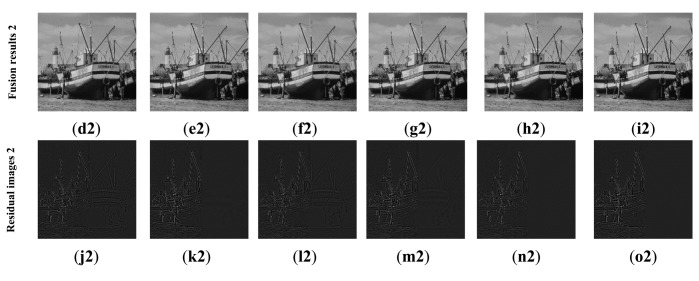
Two examples of the fusion of artificial multi-focus images. (**a1**,**a2**) Original images; (**b1**,**b2**) Images focused on right; (**c1**,**c2**) Images focused on left; (**d1**,**d2**) Wavelet results; (**e1**,**e2**) FDCT results; (**f1**,**f2**) NSCT-1 results; (**g1**,**g2**) DTCWT-1 results; (**h1**,**h2**) NSCT-2 results; (**i1**,**i2**) proposed method results; (**j1**–**o1**,**j2**–**o2**). The sharpened residual images between the six results and Figure 7b1,b2.

**Figure 8.**
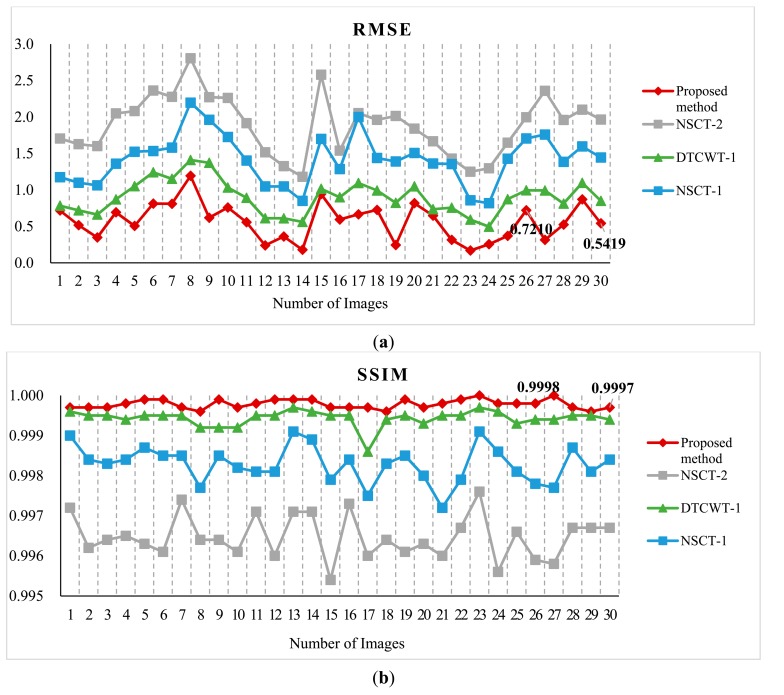
Comparison on (**a**) RMSE and (**b**) SSIM of different methods for artificial multi-focus images.

For further comparison, we experimented with more standard images. Figure 6 shows the standard test image database. The database is the standard grayscale test images that have been downloaded from [52]. The sizes of the test images are all 512 × 512. Each standard test image has been convolved with an averaging filter centered on the left part and right part, respectively. Figure 7 shows the typical examples of the fusion of artificial multi-focus images, the results of different methods and the corresponding residual images. From Figure 7, the residual images show that the proposed method results have extracted the most information from the focus region. Figure 8 shows the comparison on RMSE and SSIM of different methods for artificial multi-focus images. From Figure 8, it is seen that the results of the proposed method almost represent the referenced images with SSIM values approaching 1. From Figure 8, it is easy to find that in 30 sets of images, the SSIM and RMSE values of the proposed method results outperform other methods.

#### 4.2.2. Fusion of Images in a Noisy Environment

**Figure 9.**
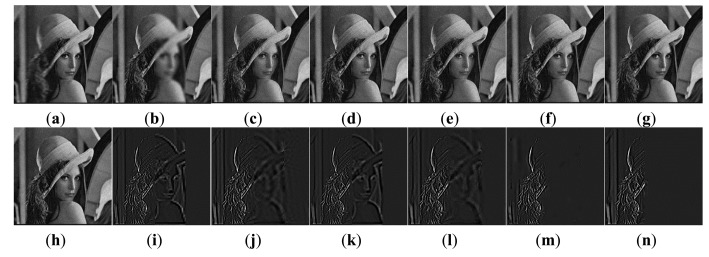
An example of the fusion for noise image. (**a**,**b**) Blurred Lena images with 5% noise; (**c**) Wavelet result; (**d**) FDCT result; (**e**) NSCT-1 result; (**f**) DTCWT-1 result; (**g**) NSCT-2 result; (**h**) proposed method result; (**i**–**n**) the sharpened residual images between the six results and Figure 9a.

**Figure 10.**
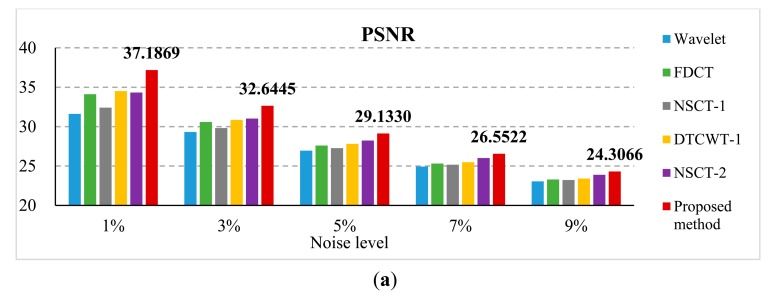

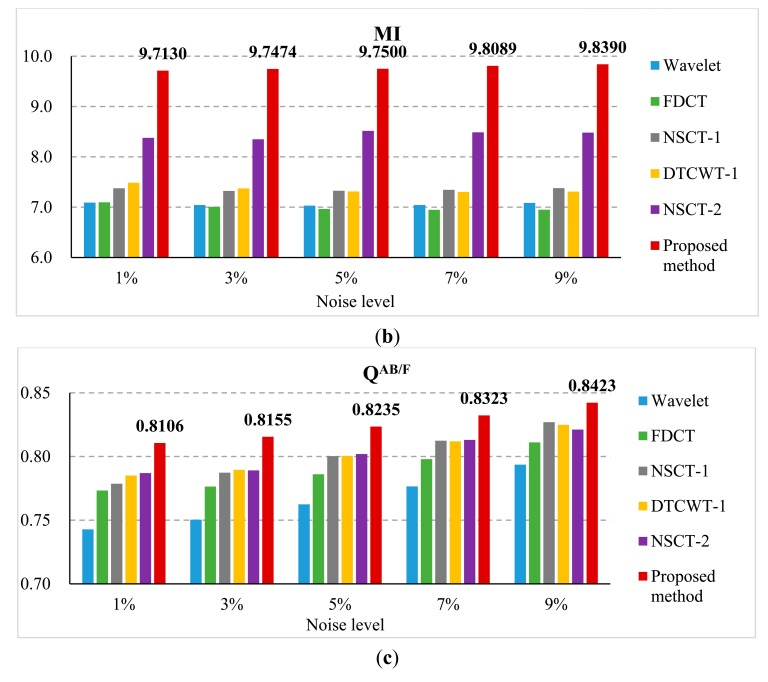
Comparison on (**a**) PSNR; (**b**) MI and (**c**) Q^AB/F^ of different methods for multi-focus image with different noise.

To evaluate the noise-robustness of the proposed framework, the second experiment is conducted on two blurred “Lena” images as shown in Figure 3b–c additionally corrupted with different Gaussian noise, with a standard deviation of 1%, 3%, 5%, 7% and 9%, respectively. Figure 9 shows the blurred images with 5% noise and the corresponding results of different methods. A quantitative evaluation comparison of the different methods for artificial images with different noise is shown in Figure 10. From Figure 10a, the PSNR values of the proposed method results are all the highest of the six methods. That is to say, in different noise environments, the results of the proposed method are closer to the referenced image than other methods. From Figure 10b,c, the MI and Q^AB/F^ values of the proposed method also outperform other methods. From the above analysis, we can observe that the proposed method provides the best performance and outperforms the other algorithms, even in noisy environments.

#### 4.2.3. Fusion of Non-Referenced Multi-Focus Images

**Figure 11.**
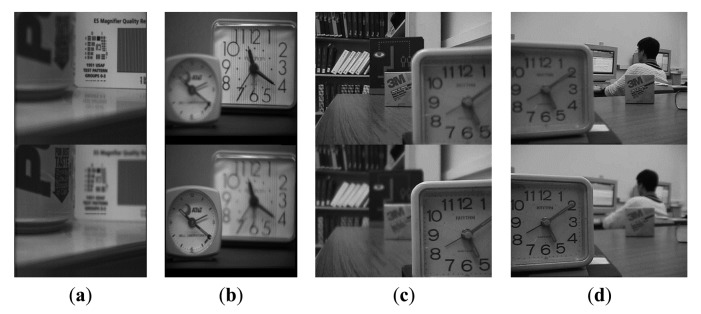
Non-referenced multi-focus image database (**top**: focused on background; **bottom**: focused on foreground). Sets of (**a**) Pepsi image; (**b**) Clock image; (**c**) Disk image; (**d**) Lab image.

**Figure 12.**
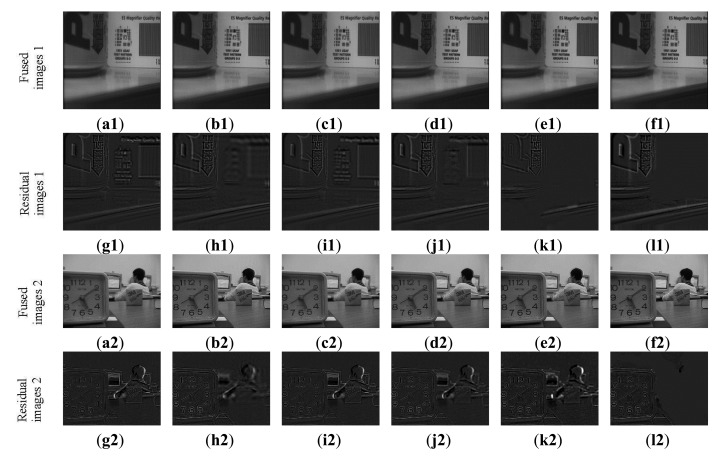
Fusion results of non-referenced multi-focus images. (**a1**,**a2**) Wavelet results; (**b1**,**b2**) FDCT results; (**c1**,**c2**) NSCT-1 results; (**d1**,**d2**) DTCWT-1 results; (**e1**,**e2**) NSCT-2 results; (**f1**,**f2**) Proposed method results; (**g1**–**l1**,**g2**–**l2**), The sharpened residual images between the six results and source image (image focused on background).

The third experiment is conducted on four sets of non-referenced multi-focus test images. These images are shown in Figure 11. The size of Figure 11a,b is 256 × 256, while the others are 480 × 640. The fusion results are shown in Figure 12a1–f1, a2–f2 and their sharpened residual images between the fused result and image focused on background are shown in Figure 12g1–l1,g2–l2. From the residual images, it is easy to find in the focused background areas that the proposed method has extracted more information than other methods and almost all of the useful information in the focus area of the source images has been transferred to the fused image.

**Figure 13.**
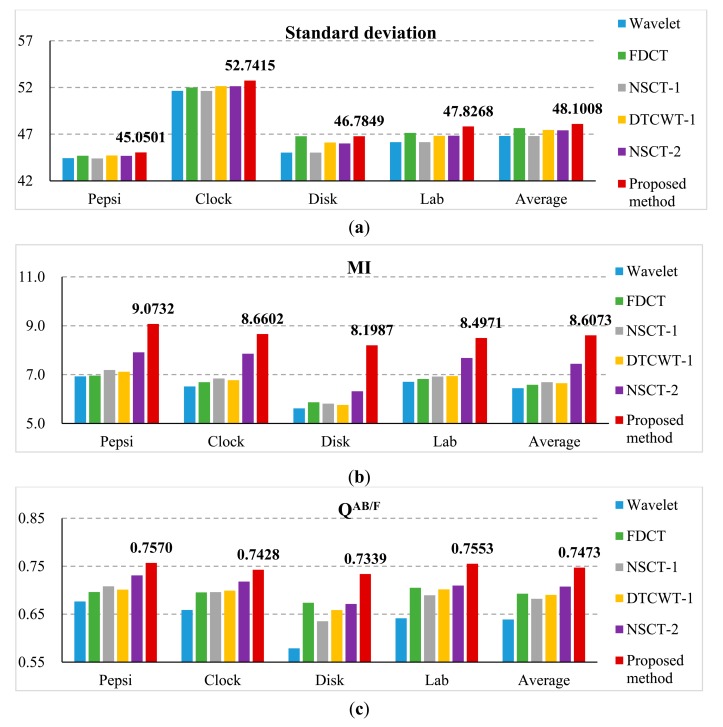
Comparison on (**a**) standard deviation; (**b**) MI and (**c**) Q^AB/F^ of different methods for non-referenced multi-focus image.

These experiments have no referenced images. Therefore, we used standard deviation, MI and Q^AB/F^ to evaluate how much clear or detailed information of the source images is transferred to the fused images. The comparison of the quantitative evaluation of different methods for non-referenced multi-focus images is shown in Figure 13. Comparing the average values of the standard deviation, MI and Q^AB/F^ of DTCWT 1 method and the proposed method, we can find that their corresponding values have been increased 1.37%, 29.50% and 8.26%, respectively. From Figure 13, the values of standard deviation, MI and Q^AB/F^ of the proposed method result are all better than those of the other methods. From the above analysis, we can also observe that the proposed scheme provides the best performance and outperforms the other algorithms.

Finally, we compare the time-consumption of the proposed method with the DTCWT- and NSCT- based methods. These methods can provide both good shift invariance and directional selectivity. A comparison of the execution times of the DTCWT-, NSCT-based methods and the proposed method for the Pepsi image sets is presented in Table 1.

The execution times are calculated by running all the codes in MATLAB R2012a on the same computer. From Table 1, it is easy to find that the NSCT-based methods (including NSCT-1 and NSCT-2 methods) are time-consuming and of high complexity, and the efficiency of the proposed method and the DTCWT-based method is higher than that of the NSCT-based methods. Although the proposed method needs a little more execution time than the DTCWT method, the fusion performance including the above qualitative and quantitative evaluations of our method have been proved better than the DTCWT method. Hence, from the overall assessments, we can conclude that the proposed method is effective and suitable for real time applications.

## 5. Conclusions

In this paper, a novel DTCWT-based multi-focus image fusion framework is proposed for VSNs. The potential advantages include: (1) DTCWT is more suitable for image fusion because its advantages such as multi-directionality and shift-invariance; (2) use of the visual contrast and SML as fusion rules to strengthen the effect of DTCWT; and (3) image block residual and consistency verification are proposed to get a fusion decision map to guide the fusion process, which not only reduces the complexity of the procedure, but also increases the reliability and robustness of the fusion results. In the experiments, extensive sets of multi-focus images, including no-referenced images, referenced images, and images with different noise, are fused by using state-of-the-art methods and the proposed framework. In the experiments with the no-referenced images, comparing with simple DTCWT-based methods, the MI and Q^AB/F^ values of the proposed method have been increased 29.50% and 8.26%, respectively. The experimental results have shown the superior performance of the proposed fusion framework. In the future, we plan to design a pure C++ platform to reduce the time cost and validate our framework in multi-sensor image fusion problems like infrared and visible light image fusion.

## Figures and Tables

**Table 1.** Execution time of different methods for Pepsi images.

**Fusion Scheme**	**NSCT-1**	**DTCWT**	**NSCT-2**	**Proposed Method**
Execution time (s)	14.4024	0.3273	32.6268	3.3020
